# Platelet-Rich Plasma in Recurrent Pregnancy Loss: Toward a Precision Medicine Framework for Biologically Guided Patient Selection

**DOI:** 10.3390/medsci14050576

**Published:** 2026-09-17

**Authors:** Sofoklis Stavros, Anastasios Potiris, Maria Anastasia Daskalaki, Stefanos Dafopoulos, Efthalia Moustakli, Theodoros Karampitsakos, Dimos Sioutis, Konstantinos Dafopoulos, Nikolaos Thomakos, George Daskalakis, Peter Drakakis

**Affiliations:** 1 Third Department of Obstetrics and Gynecology, University General Hospital “ATTIKON”, Medical School, National and Kapodistrian University of Athens, 12462 Athens, Greece; theokarampitsakos@hotmail.com (T.K.); dsioutis@gmail.com (D.S.); pdrakakis@hotmail.com (P.D.); 2 Department of Obstetrics and Gynecology, ‘Alexandra’ General Hospital, National and Kapodistrian University of Athens, 80 Vasilissis Sofias Avenue, 11528 Athens, Greece; anastasia.daskalaki00@gmail.com (M.A.D.); nthomakos@med.uoa.gr (N.T.); gdaskalakis@yahoo.com (G.D.); 3 University General Hospital of Patras, 26504 Rio, Greece; stefanosntf2001@gmail.com; 4 Department of Nursing, School of Health Sciences, University of Ioannina, 4th Kilometer National Highway Str. Ioannina-Athens, 45500 Ioannina, Greece; ef.moustakli@uoi.gr; 5 Department of Obstetrics and Gynaecology, Faculty of Medicine, School of Health Sciences, University of Thessaly, 41110 Larissa, Greece; kdafop@uth.gr

**Keywords:** recurrent pregnancy loss, platelet-rich plasma, endometrial receptivity, precision medicine, maternal–fetal interface, immunomodulation, angiogenesis, regenerative medicine

## Abstract

Recurrent pregnancy loss (RPL), a multifactorial condition in reproductive medicine, affects approximately 1–3% of couples. Increasing evidence implicates endometrial dysfunction, immune dysregulation, impaired angiogenesis, and oxidative stress (OS) in its pathophysiology, highlighting the need for biologically targeted therapeutic strategies. In this context, platelet-rich plasma (PRP) has emerged as a regenerative approach with the potential to modulate these underlying mechanisms. This narrative review critically evaluates the biological rationale and current clinical evidence supporting PRP as an adjunctive treatment for RPL while proposing a precision medicine framework for biologically guided patient selection. A comprehensive narrative synthesis of the literature was performed, focusing on studies investigating PRP in RPL and related reproductive conditions, including thin endometrium and recurrent implantation failure (RIF). Mechanistically, PRP promotes angiogenesis through vascular endothelial growth factor (VEGF)-mediated pathways, enhances endometrial regeneration by stimulating stromal and epithelial cell proliferation, and modulates immune responses by promoting regulatory T-cell activity while attenuating pro-inflammatory signaling. In addition, PRP-derived extracellular vesicles (EVs) and microRNAs may contribute to the post-transcriptional and epigenetic regulation of implantation-related genes, including leukemia inhibitory factor (LIF) and HOXA10. Clinical studies in RIF and thin endometrium populations suggest that PRP may improve endometrial thickness, implantation, and clinical pregnancy outcomes; however, these findings constitute indirect evidence for RPL. Direct RPL-specific evidence remains very limited and is insufficient to establish a reduction in miscarriage or an improvement in live birth. These biological features instead provide a rationale for investigating PRP in defined patient subgroups characterized by impaired endometrial receptivity, defective angiogenesis, immune dysregulation, or unexplained RPL with suspected endometrial dysfunction. Accordingly, we propose that future clinical investigation of PRP should move beyond empirical use toward biomarker-informed, precision reproductive medicine strategies. Although PRP represents a biologically plausible and promising adjunctive therapy, robust randomized controlled trials incorporating standardized PRP protocols and biologically stratified patient populations are required before its routine clinical use can be recommended.

## 1. Introduction

One of the most complex, multifactorial, and distressing challenges in the field of reproductive medicine for both healthcare professionals and couples is recurrent pregnancy loss (RPL). RPL is defined as the occurrence of two or more pregnancy losses, which do not need to be consecutive [1,2]. RPL affects approximately 1–3% of couples attempting to conceive, and despite advances in assisted reproductive technologies (ART) and molecular diagnostics, nearly half of RPL cases remain unexplained after medical evaluation [3,4]. Common causal factors of RPL include chromosomal abnormalities, uterine anatomical defects, endocrine disorders, thrombophilias, and autoimmune conditions [4]. However, a growing body of evidence highlights the critical role of the endometrium in successful apposition, adhesion, and implantation [5,6]. Implantation and placentation require finely coordinated processes, including decidualization, immune tolerance, angiogenesis, extracellular matrix remodeling, and oxidative balance [7,8]. Disruption of these finely tuned cyclical events may predispose individuals to implantation failure or early miscarriage, even in the presence of euploid embryos [9].

Decidualization, characterized by stromal cell differentiation and progesterone responsiveness, plays a critical role in embryo selection and trophoblast invasion. Dysregulated expression of HOXA10, LIF, integrins, and cytokine-related genes has been reported in women with RPL [10,11,12]. This dysregulation may lead to a defective window of implantation and ultimately result in abnormal placentation. Defective spiral artery remodeling, along with abnormal gene expression, contributes to impaired early placentation and increases the risk of early pregnancy loss [13,14].

Another key contributor to the pathophysiology of RPL is dysregulation of the immune response at the maternal–fetal interface [15]. Early pregnancy involves a finely balanced process, beginning with a controlled pro-inflammatory phase that facilitates implantation, followed by a shift toward an immune-tolerant environment necessary for maintaining gestation [16,17]. This dynamic equilibrium depends on coordinated interactions between macrophages, dendritic cells, regulatory T cells, and uterine natural killer cells [18,19]. Disruption of this balance, particularly through excessive or poorly regulated inflammation, may impair implantation and compromise early pregnancy development.

**Figure 1:**
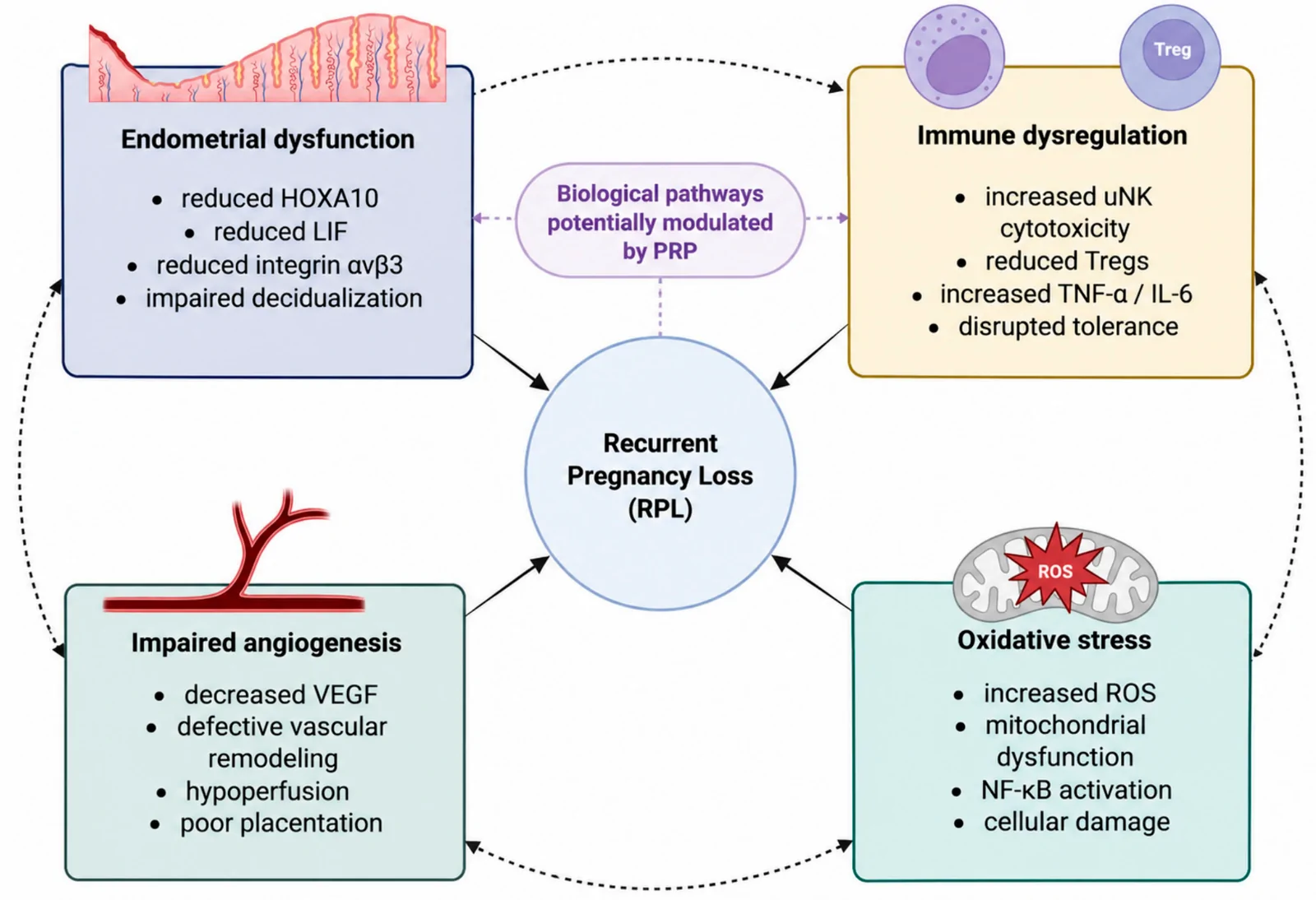
Proposed pathophysiological mechanisms implicated in recurrent pregnancy loss (RPL) and biological pathways potentially modulated by PRP. Endometrial dysfunction, immune dysregulation, impaired angiogenesis, and oxidative stress represent biological processes associated with RPL, whereas their modulation by PRP is based predominantly on mechanistic, experimental, and indirect reproductive evidence. The illustrated relationships should therefore be interpreted as biologically plausible and hypothesis-generating rather than as established causal effects of PRP in women with RPL.

RPL is increasingly linked to oxidative stress (OS) and mitochondrial dysfunction. Excess production of reactive oxygen species (ROS) can disrupt cellular integrity, impair decidualization, and interfere with angiogenic processes [20,21]. Simultaneously, reduced antioxidant capacity within the endometrium may amplify inflammatory signaling pathways [22]. Figure 1 illustrates the complex relationship between OS, inflammation, and abnormal vascular remodeling, a pathophysiological triad that may account for a significant proportion of unexplained miscarriage [23,24]. This knowledge has fueled the growing interest in novel, biologically targeted, and regenerative therapeutic approaches aimed at restoring intrauterine adequacy and maternal–fetal immune balance.

PRP is an autologous blood-derived product obtained by centrifugation procedures that concentrate platelets in a reduced plasma volume. Although preparation protocols vary, PRP generally contains platelet concentrations several times higher than basal levels in peripheral blood. Upon activation, platelets release a wide range of bioactive molecules, including VEGF, platelet-derived growth factor (PDGF), transforming growth factor-β (TGF-β), insulin-like growth factor-1 (IGF-1), epidermal growth factor (EGF), and basic fibroblast growth factor (bFGF) [25,26]. In addition, PRP contains cytokines, chemokines, adhesion molecules, and EVs, which collectively regulate cell proliferation, migration, angiogenesis, and inflammation [26,27].

PRP has been used in different medical specialties, including orthopedics, dermatology, urology, and plastic surgery, due to its regenerative and immunomodulatory properties. More specifically, in gynecology, PRP has been used for urinary incontinence, as an experimental therapy for thin endometrium and Asherman syndrome, and for ovarian rejuvenation in women with diminished ovarian reserve [27,28,29,30,31]. Its autologous nature minimizes the risk of immunogenic and infectious complications, making it an attractive therapeutic option in sensitive clinical settings such as early pregnancy. Accordingly, its application in RPL is biologically plausible, as it targets key underlying mechanisms including impaired endometrial receptivity, vascular insufficiency, and immune dysregulation.

Theoretically, PRP may enhance endometrial regeneration by stimulating stromal cell proliferation and promoting re-epithelialization [31,32]. Its angiogenic growth factors may support neovascularization and improve microvascular perfusion, both of which are essential for early placental development [33]. In addition, PRP-derived cytokines may modulate inflammatory signaling and help establish a more favorable immunological microenvironment [26,34]. Despite this strong biological rationale, the use of PRP in RPL remains at an early and largely experimental stage. Current clinical evidence is limited, heterogeneous, and primarily derived from small patient cohorts.

Importantly, the current clinical application of PRP in RPL has largely been empirical, with limited consideration of the biological heterogeneity underlying recurrent pregnancy loss. Given the multifactorial nature of RPL, it is unlikely that PRP represents a universally effective intervention. Instead, its therapeutic potential may be confined to biologically defined patient subgroups characterized by impaired endometrial receptivity, defective angiogenesis, immune dysregulation, or oxidative stress. These observations highlight the need to move beyond empirical treatment toward the investigation of biologically guided patient stratification within a precision reproductive medicine framework.

Precision and regenerative approaches to PRP have previously been discussed in reproductive medicine, particularly in the context of RIF and endometrial dysfunction. The specific contribution of the present review is to critically examine this concept in the context of RPL by integrating mechanistic evidence at the endometrium–maternal–fetal interface with a population-specific appraisal of the available clinical evidence. In particular, we distinguish direct evidence derived from RPL populations from indirect evidence derived from RIF, thin endometrium, and other infertility cohorts and prioritize miscarriage and live birth over surrogate endometrial or implantation outcomes when evaluating clinical relevance to RPL. Building on this distinction, we propose a hypothesis-generating framework for future biological stratification based on endometrial receptivity, angiogenesis, immune regulation, and oxidative stress. This framework is intended to inform future biomarker-driven clinical research rather than current clinical patient selection or treatment decisions.

## 2. Literature Search Strategy

A narrative literature review was conducted to evaluate the role of PRP in RPL and related reproductive conditions. The relevant literature was identified through searches of PubMed, Scopus, and Web of Science. The literature search was initially completed on 30 June 2026 and subsequently updated on 29 August 2026 before manuscript revision. The search strategy combined Medical Subject Headings (MeSH), where applicable, and free-text terms, including “recurrent pregnancy loss,” “recurrent miscarriage,” “platelet-rich plasma,” “PRP,” “endometrial receptivity,” “implantation failure,” “angiogenesis,” and “immunomodulation.” Boolean operators (“AND,” “OR”) were used to combine terms and refine the searches.

Studies were considered eligible if they were published in English, available as full-text articles, and relevant to the clinical application or biological mechanisms of PRP in RPL or related reproductive conditions, including recurrent implantation failure and thin endometrium. Both human clinical studies and experimental studies providing mechanistic evidence relevant to PRP and endometrial function were considered. Studies were excluded if they were not published in English, lacked sufficient methodological detail, were outside the scope of the review, or were conference abstracts without full-text availability. Titles and abstracts were initially screened for relevance, followed by full-text evaluation of potentially relevant articles. Reference lists of included articles were also manually screened to identify additional relevant publications.

As this review was designed as a narrative synthesis rather than a systematic review, a formal systematic search protocol was not prospectively established, and record-level counts of identified, screened, included, and excluded studies, together with individual reasons for exclusion, were not prospectively recorded. Accordingly, a PRISMA-style study-flow process and formal risk-of-bias assessment were not applied. The absence of prospectively documented complete search strings and record-level screening data limits the reproducibility of the literature search and should be considered an inherent methodological limitation of this narrative review. Instead, studies were selected based on their relevance, methodological clarity, and contribution to understanding the mechanistic rationale or clinical applicability of PRP. Particular emphasis was placed on studies with clearly defined patient populations, reported clinical outcomes, and adequately described PRP protocols. For the clinical evidence synthesis, RPL-specific studies were considered direct evidence, whereas studies involving recurrent implantation failure, thin endometrium, or other infertility populations were considered indirect evidence relevant to the potential reproductive effects of PRP. This approach was intended to provide a structured and clinically meaningful synthesis of the available evidence while acknowledging the inherent limitations of a narrative review design.

## 3. PRP and Endometrial Receptivity

Endometrial receptivity refers to the transient period during which the endometrium acquires the morphological, molecular, and functional characteristics required for embryo adhesion and implantation [35]. This interval occurs during the middle of the secretory phase and is regulated by endocrine, molecular, and cellular signals [36]. The receptive endometrium is characterized by coordinated changes in epithelial morphology, stromal differentiation, gene expression, vascular development, and immune signaling [37,38,39]. In women with RPL, several abnormalities affecting endometrial receptivity have been described, including altered expression of implantation-related genes, defective stromal decidualization, impaired vascularization, and dysregulated inflammatory signaling. These alterations provide a biologically plausible rationale for investigating the potential therapeutic effects of PRP. Importantly, these alterations suggest that any potential benefit of PRP may differ according to the underlying endometrial phenotype rather than being uniform across the heterogeneous RPL population.

### 3.1. PRP and Endometrial Regeneration

PRP contains a variety of growth factors, including PDGF, EGF, IGF-1, and TGF-β [25,26]. Upon activation, platelets release these factors into the surrounding tissue, where they promote cellular proliferation, migration, and differentiation [40,41]. In the endometrium, these effects involve both epithelial and stromal cells [42]. Epithelial cells, which line the endometrial cavity, represent the first point of contact with the implanting embryo, while stromal cells provide structural support and undergo transformation during preparation for implantation.

PDGF and IGF-1 activate intracellular signaling pathways, including PI3K/AKT and MAPK/ERK, which regulate cell survival, proliferation, and differentiation [43,44,45]. Activation of these pathways promotes the expansion of endometrial cell populations and contributes to the restoration of a structurally and functionally adequate endometrial lining [31,46].

In addition, PRP may enhance the regenerative capacity of the endometrium by activating stem and progenitor cells within the basal layer, which are responsible for cyclic endometrial renewal following menstruation [47,48,49]. The growth factors present in PRP may stimulate these cells, promote tissue repair, and improve both endometrial thickness and quality in patients with endometrial dysfunction.

From a clinical perspective, these mechanisms support a potential role for PRP in patients with thin endometrium or impaired endometrial regeneration, whereas the relevance of PRP in patients without structural or functional endometrial abnormalities remains uncertain.

### 3.2. PRP and Regulation of Endometrial Gene Expression

Several molecular factors, including integrin αvβ3, leukemia inhibitory factor (LIF), and HOXA10, play critical roles in implantation [12,50]. Women with RPL have been shown to have altered expression of these markers, suggesting a biological basis for impaired endometrial receptivity. PRP-derived growth factors may influence the expression of these genes by activating intracellular signaling pathways that regulate transcriptional activity. Specifically, EGF and IGF-1 activate receptor tyrosine kinases that trigger the PI3K/AKT and MAPK/ERK pathways, which in turn regulate transcription factors essential for endometrial differentiation [45,51].

The progesterone-regulated transcription factor HOXA10 plays an essential role in establishing endometrial receptivity for implantation. It regulates genes involved in immune signaling, stromal differentiation, and cell adhesion [52,53]. It facilitates embryo adhesion by increasing the expression of integrins on the uterine epithelial surface [12,54,55]. Experimental evidence suggests that PRP-derived growth factors may improve the molecular profile of endometrial receptivity by upregulating HOXA10 expression [56,57,58].

Through the JAK/STAT signaling pathway, LIF, a cytokine essential for implantation, promotes embryo adhesion and maternal–fetal communication. Reduced LIF expression has been associated with implantation failure and early pregnancy loss [59,60]. PRP-derived factors have been proposed to modulate cytokine networks and potentially influence LIF-associated signaling, although this mechanism remains insufficiently established in the context of RPL. Although HOXA10 and LIF are established components of endometrial receptivity and altered expression has been associated with reproductive dysfunction, direct evidence that intrauterine PRP restores their expression or improves RPL outcomes through these pathways is lacking. Their proposed involvement in the response to PRP should therefore be considered mechanistic and hypothesis-generating.

These observations provide a biological rationale for investigating PRP in RPL subgroups characterized by molecular abnormalities of endometrial receptivity; however, whether such abnormalities predict response to PRP remains unknown, and no validated biomarkers are currently available for clinical patient selection.

### 3.3. PRP and Angiogenesis in the Endometrium

Adequate vascular development within the endometrium is essential to meet the metabolic and oxygen demands of the implanted embryo. During the mid-secretory phase, the endometrial microvasculature undergoes dynamic remodeling, enhancing tissue perfusion and facilitating nutrient exchange [61,62]. Impaired vascularization has been associated with implantation failure and early pregnancy loss.

PRP contains high concentrations of angiogenic factors, including VEGF and bFGF. VEGF plays a key role in endothelial cell proliferation, migration, and survival [63]. Upon binding to its receptors (VEGFR-1 and VEGFR-2), it activates intracellular signaling pathways such as PI3K/AKT and nitric oxide synthase (eNOS), promoting endothelial cell survival and vasodilation [63,64,65]. Through these mechanisms, PRP may enhance the formation of a dense and functional microvascular network within the receptive endometrium and may improve microcirculation by increasing oxygen and nutrient delivery [31,66,67]. In addition, improved vascularization may contribute to stabilization of the endometrial environment during the early stages of implantation.

These angiogenic mechanisms provide a biological rationale for investigating PRP in RPL subgroups characterized by suspected endometrial vascular insufficiency; however, whether vascular abnormalities predict response to PRP remains unknown.

### 3.4. Regulation of Inflammatory Response

Implantation is accompanied by a controlled inflammatory response within the endometrium. However, excessive or persistent inflammation may disrupt endometrial receptivity and compromise embryo implantation [68]. PRP contains a variety of cytokines and growth factors capable of modulating inflammatory signaling pathways. Platelet-derived factors have been shown to regulate the activity of NF-κB, a key transcription factor involved in inflammatory responses. Inhibition of NF-κB activation may reduce the production of pro-inflammatory cytokines such as tumor necrosis factor-α (TNF-α) and interleukin-6 (IL-6), helping maintain a balanced inflammatory environment within the endometrium [69,70,71]. By promoting a controlled inflammatory state rather than excessive immune activation, PRP may support the molecular conditions necessary for embryo adhesion and implantation.

These immunomodulatory properties provide a biological rationale for investigating PRP in RPL subgroups characterized by inflammatory or immune dysregulation; however, whether such patients derive clinical benefit from PRP remains unproven and requires prospective evaluation.

### 3.5. Extracellular Matrix Remodeling

Successful embryo attachment requires coordinated remodeling of the extracellular matrix (ECM) within the endometrium. ECM components provide structural support and regulate cell adhesion and migration [7,72,73]. Matrix metalloproteinases (MMPs), particularly MMP-2 and MMP-9, participate in ECM degradation and remodeling during implantation [74,75]. The growth factors present in PRP influence the remodeling of the ECM by regulating the expression of MMPs and their inhibitors (TIMPs) [76]. Through this balanced regulation, PRP may facilitate structural modifications of the endometrial surface that enhance embryo adhesion while preserving tissue integrity [76,77]. Furthermore, integrins expressed on the epithelial surface of the endometrium mediate the physical interaction between the embryo and the uterine lining. PRP-derived signaling pathways may increase integrin expression, thereby enhancing the adhesive capacity of the receptive endometrium [51,68,78].

Overall, these mechanisms provide a biological rationale for investigating PRP in RPL subgroups characterized by endometrial or implantation-related dysfunction; however, their clinical relevance and ability to predict response to PRP remain unproven.

## 4. PRP at the Maternal–Fetal Interface

The maternal–fetal interface represents a highly specialized microenvironment where maternal tissues interact with the developing fetus to support implantation, placental formation, and early embryonic development [79]. This interface consists primarily of decidualized endometrial stromal cells, trophoblast cells derived from the embryo, and a diverse population of maternal immune cells [80,81]. Successful pregnancy requires tightly regulated communication between these components to coordinate trophoblast invasion, vascular remodeling, immune tolerance, and inflammatory balance.

PRP has emerged as a potential adjunct therapeutic agent by delivering a concentrated mixture of growth factors, cytokines, chemokines, and EVs. Proposed mechanisms include modulation of decidualization, trophoblast function, angiogenic signaling, and immune homeostasis. Table 1 summarizes the proposed biological effects of PRP relevant to the endometrium and maternal–fetal interface, together with the evidence basis supporting each mechanism.

The mechanistic evidence discussed below derives from different sources and should therefore be interpreted according to its evidentiary context. Direct human reproductive evidence is available for some effects of PRP on endometrial function; however, several proposed mechanisms, particularly those involving immune regulation, OS, EVs, and miRNAs, are supported predominantly by in vitro, experimental, non-reproductive, or extrapolated evidence. These mechanisms should therefore be considered biologically plausible and hypothesis-generating rather than established effects of intrauterine PRP in women with RPL [82,83]. Accordingly, the evidence classifications presented in Table 1 indicate the source and context of the available evidence and should not be interpreted as formal certainty or risk-of-bias ratings. Direct evidence demonstrating these mechanisms following intrauterine PRP administration in women with RPL remains limited or absent, as indicated for each mechanism.

**Table 1:** Proposed biological effects of platelet-rich plasma relevant to the endometrium and maternal–fetal interface and the corresponding evidence basis.

Biological Process	Target Cells	Key PRP Factors	Molecular Pathways	Proposed Functional Effects Relevant to Early Pregnancy	Evidence Basis	References
Decidualization	Endometrial stromal cells	TGF-β, PDGF, IGF-1, EGF	PI3K/AKT, MAPK/ERK, cAMP-dependent signaling	Biologically plausible modulation of stromal-cell differentiation and decidual function; direct PRP-specific evidence remains limited	Mechanistic human endometrial/decidual evidence; direct PRP-specific and RPL-specific evidence lacking	[84,85]
Endometrial regeneration and tissue remodeling	Endometrial epithelial and stromal cells	PDGF, EGF, TGF-β, IGF-1	MAPK/ERK, PI3K/AKT and growth-factor-associated signaling	Proposed promotion of cellular proliferation and tissue remodeling through growth-factor-mediated signaling	Indirect mechanistic evidence from uterine/endometrial and general growth-factor biology; direct PRP-specific and RPL-specific evidence lacking	[43,44,45]
Trophoblast invasion	Trophoblast cells	PRP-derived EVs and associated bioactive cargo	EV-mediated intercellular signaling	Potential modulation of cellular processes relevant to trophoblast migration and invasion	Indirect mechanistic evidence based on PRP-derived EV biology; direct evidence for trophoblast invasion following intrauterine PRP in RPL is lacking	[86,87]
Extracellular matrix degradation	Trophoblasts, stromal cells	MMP-2, MMP-9	ECM remodeling pathways	Potential modulation of ECM remodeling and processes relevant to trophoblast invasion	Indirect reproductive and mechanistic evidence; direct evidence for PRP-mediated ECM remodeling in women with RPL is lacking	[88,89]
Angiogenesis	Endothelial cells	VEGF	VEGF/VEGFR signaling	Biologically plausible promotion of endothelial proliferation and angiogenic signaling through VEGF-related pathways	Established VEGF/VEGFR angiogenic biology; direct evidence for PRP-induced angiogenesis in women with RPL is lacking	[64]
Vascular maturation	Pericytes, smooth muscle cells	PDGF-BB	PDGF/PDGFR signaling	Potential support of mural-cell recruitment and vascular stabilization through PDGF-mediated signaling	Experimental and general vascular evidence for PDGF-mediated mural-cell recruitment; direct PRP-specific, reproductive, and RPL-specific evidence lacking	[90,91]
Immune tolerance	Tregs	TGF-β	TGF-β/SMAD signaling	Biologically plausible modulation of Treg differentiation and immune-tolerance pathways through TGF-β signaling	Pregnancy-related and general immunological evidence for TGF-β/Treg regulation; direct evidence for PRP-induced Treg modulation in women with RPL is lacking	[92,93]
Regulation of uterine NK cells	uNK cells	Cytokines and growth factors	Cytokine-mediated immune signaling	Potential modulation of uNK-cell functions involved in immune regulation and vascular remodeling during pregnancy	Human pregnancy-related evidence for uNK biology and cytokine regulation; direct evidence that intrauterine PRP modulates uNK cells in women with RPL is lacking	[94,95]
Macrophage polarization	Decidual macrophages	PRP-derived bioactive factors	M1/M2 polarization-associated signaling	Potential modulation of macrophage polarization toward a reparative M2-associated phenotype	Human pregnancy evidence supports the relevance of decidual macrophage polarization, while PRP-mediated M1/M2 modulation is supported by experimental evidence; direct evidence following intrauterine PRP in women with RPL is lacking	[96,97]
Anti-inflammatory signaling	Immune and endometrial cells	PRP-derived cytokines and growth factors	NF-κB and inflammation-associated signaling	Potential attenuation of pro-inflammatory signaling and modulation of the local inflammatory response	PRP-specific mechanistic evidence supports anti-inflammatory effects in broader biological contexts; direct human reproductive and RPL-specific evidence is lacking	[26]
OS regulation	Endometrial and reproductive cells	PRP-derived bioactive factors	Nrf2-mediated antioxidant signaling	Potential enhancement of cellular antioxidant defenses and attenuation of oxidative damage	PRP-specific experimental evidence supports Nrf2-mediated antioxidant effects in non-reproductive cells; direct evidence in human endometrium and women with RPL is lacking	[98,99]
Epigenetic and microRNA signaling	Endometrial and maternal–fetal interface cells	PRP-derived bioactive factors and EV-associated cargo	miRNA-mediated gene regulation	Hypothesized modulation of gene-regulatory pathways relevant to endometrial function and pregnancy maintenance	Human RPL evidence supports altered miRNA expression in RPL, while PRP-derived EVs provide a biologically plausible source of regulatory molecular cargo; a direct PRP–EV/miRNA mechanism in the endometrium or RPL has not been demonstrated.	[86,87,100]
Mitochondrial support	Endometrial cells	PRP-derived bioactive factors	Mitochondrial homeostasis and cellular stress-response pathways	Hypothesized support of mitochondrial homeostasis and cellular function in the endometrium	Indirect evidence from reviews of PRP in thin endometrium and endometrial receptivity; direct experimental evidence for PRP-mediated mitochondrial effects in human endometrium or RPL is lacking	[31,101]

Notably, these proposed mechanisms largely concern disturbances at the level of the endometrium and maternal–fetal interface. This provides a biological rationale for investigating PRP in RPL subgroups characterized by implantation-related or microenvironmental dysfunction, while its relevance to non-endometrial causes of RPL remains uncertain.

### 4.1. PRP and Modulation of Decidualization

Decidualization is the process by which endometrial stromal cells differentiate into specialized decidual cells under the influence of progesterone and cyclic adenosine monophosphate (cAMP) [82]. This transformation is essential for embryo implantation and early pregnancy maintenance. Decidual cells regulate trophoblast invasion, produce critical cytokines and growth factors, and contribute to immune tolerance. In women with RPL, impaired decidualization has been associated with abnormal expression of genes involved in implantation and tissue remodeling [83].

The potential effects of PRP on endometrial decidualization may involve several molecular mechanisms. Growth factors such as TGF-β, PDGF, and IGF-1 are known to stimulate stromal cell proliferation, and differentiation [84]. These factors activate intracellular signaling cascades, including the PI3K/AKT and MAPK pathways, which regulate cell survival, proliferation and differentiation [102,103]. Activation of these pathways promotes the transition of stromal fibroblasts into functional decidual cells capable of supporting embryo implantation.

Additionally, PRP may influence the expression of key decidualization markers such as prolactin (PRL) and insulin-like growth factor binding protein-1 (IGFBP-1) [85]. These molecules are hallmarks of decidual transformation and play important roles in regulating trophoblast invasion and maternal–fetal communication. Through potential modulation of these genes, PRP may influence the functional competence of the decidua in women with compromised endometrial receptivity [58,104].

These findings provide a biological rationale for investigating PRP in RPL subgroups characterized by defective decidualization; however, whether this phenotype predicts response to PRP remains unknown.

### 4.2. PRP and Trophoblast Invasion and Placentation

Trophoblast invasion into the maternal decidua and myometrium is a critical step in establishing early placentation. Extravillous trophoblasts (EVTs) migrate into maternal tissues and participate in the remodeling of the spiral arteries. This process transforms spiral arteries from high-resistance vessels into low-resistance vessels capable of supplying adequate blood flow to the developing placenta [105,106]. Insufficient trophoblast invasion and defective spiral artery remodeling are key mechanisms implicated in early pregnancy loss [107].

PRP contains various growth factors that may enhance trophoblast migration and invasion. Epidermal growth factor (EGF), VEGF, and fibroblast growth factor (FGF) are known to stimulate trophoblast proliferation and motility [108]. These factors activate tyrosine kinase receptors, leading to the activation of intracellular signaling pathways, such as MAPK/ERK and PI3K/AKT, which regulate cytoskeletal dynamics, cell adhesion, and extracellular matrix degradation [109,110].

MMPs, particularly MMP-2 and MMP-9, play a crucial role in trophoblast invasion by degrading components of the extracellular matrix in the decidua [88]. The cytokines and growth factors in PRP appear to enhance the expression and/or activation of these enzymes, facilitating the controlled invasion of trophoblasts [48,77]. At the same time, regulatory mechanisms, such as TIMPs, maintain the necessary balance, preventing excessive invasion [77,89]. Through this finely tuned balance, PRP may support normal trophoblast invasion while maintaining tissue integrity.

This mechanistic rationale provides a biological basis for investigating PRP in RPL subgroups characterized by impaired placentation; however, its clinical relevance remains uncertain, and validated markers for identifying such patients are currently lacking.

### 4.3. PRP in Angiogenesis and Vascular Remodeling

Angiogenesis is essential for establishing an adequate uteroplacental circulation. During early pregnancy, extensive vascular remodeling occurs within the decidua and placenta to ensure sufficient oxygen and nutrient delivery to the developing embryo. Impaired angiogenic signaling has been associated with RPL and other placental disorders [111].

PRP is rich in angiogenic growth factors, including VEGF, PDGF, and basic fibroblast growth factor (bFGF). VEGF plays a central role in endothelial cell proliferation, migration, and survival, promoting the formation of new blood vessels within the endometrium and decidua. Binding of VEGF to its receptors (VEGFR-1 and VEGFR-2) activates downstream signaling pathways such as PI3K/AKT and endothelial nitric oxide synthase (eNOS)-mediated signaling, which enhance endothelial cell survival and vascular permeability [64].

PDGF contributes to the maturation and stabilization of newly formed vessels by recruiting smooth muscle cells [90,91]. Together with VEGF and FGF, these factors may facilitate the development of a functional microvascular network capable of supporting placental growth [90,112]. Improved vascularization may also enhance oxygen delivery and reduce local hypoxia, which is known to trigger inflammatory responses and OS.

These mechanisms provide a biological rationale for investigating PRP in RPL subgroups characterized by vascular or angiogenic impairment; however, whether such abnormalities predict response to PRP remains unknown.

### 4.4. PRP and Immunomodulation

Immune tolerance toward the semi-allogeneic fetus is a hallmark of successful pregnancy. At the maternal–fetal interface, immune cells must balance defense against pathogens with tolerance toward fetal antigens [113]. A disruption of this balance can lead to excessive inflammatory reactions, jeopardizing implantation and early fetal development [114].

PRP may help regulate the immune response through its effects on cytokine signaling and immune cell function. Transforming growth factor-β (TGF-β), which is present in PRP, is a key immunoregulatory cytokine that promotes the differentiation of regulatory T cells (Tregs), which are critical for maintaining immune tolerance during pregnancy [92,93]. Increased Treg activity suppresses pro-inflammatory T-cell responses and contributes to the formation of a supportive immune environment in the decidua [115]. However, these observations primarily provide a biological rationale, and direct evidence that intrauterine PRP enhances Treg-mediated tolerance in women with RPL is lacking.

In addition, PRP may influence the activity of uterine natural killer cells (uNKs), which constitute the predominant population of immune cells during the early stages of pregnancy [94,116]. Unlike peripheral NK cells, uNKs primarily play a regulatory role, contributing to implantation and vascular remodeling through the production of cytokines and angiogenic factors [94,95]. Although limited human reproductive evidence suggests that intrauterine PRP may influence the endometrial immune environment, direct evidence demonstrating modulation of uNK-cell function by PRP in women with RPL remains unavailable [116].

Macrophages in the decidua also contribute to immune regulation and tissue remodeling. Depending on the local cytokine environment, they can differentiate into pro-inflammatory (M1) or anti-inflammatory (M2) phenotypes [96,117]. Experimental evidence suggests that PRP may promote polarization toward the M2 phenotype, which is associated with tissue repair, angiogenesis, and immune tolerance [97]. However, this effect has not been directly established in the human endometrium or in women with RPL.

These immunomodulatory mechanisms remain biologically plausible but incompletely validated in human reproductive tissues and provide a rationale for investigating PRP in RPL subgroups characterized by immune dysregulation. However, whether immunological profiles predict response to PRP remains unknown, and standardized immunological biomarkers for PRP patient selection have not been clinically validated.

### 4.5. PRP and OS Modulation

Inflammation and OS are closely interrelated processes that may contribute to pregnancy loss. Excessive production of ROS can damage cellular structures, disrupt mitochondrial function, and activate inflammatory signaling pathways, including nuclear factor kappa B (NF-κB) signaling [118,119,120]. At the same time, women with RPL often exhibit elevated markers of OS in both the endometrium and the peripheral circulation [121].

Although these findings support a role for OS in RPL, direct evidence that intrauterine PRP modifies oxidative stress in the human endometrium or in women with RPL remains lacking. Experimental and non-reproductive evidence suggests that PRP may exert protective effects by modulating inflammatory pathways and enhancing antioxidant defense. Platelet-derived growth factors have been reported to suppress NF-κB activation, thereby reducing the production of pro-inflammatory cytokines such as TNF-α and IL-6 [26]. Experimental studies also suggest that PRP can stimulate the expression of antioxidant enzymes that neutralize ROS and restore cellular redox balance [98,99].

Furthermore, potential effects of PRP on mitochondrial biogenesis and cellular energy metabolism have been proposed on the basis of experimental and regenerative-medicine evidence [31,101]. Adequate mitochondrial function is critical for cell proliferation and differentiation during the early stages of pregnancy. However, whether intrauterine PRP preserves mitochondrial function or reduces OS at the human maternal–fetal interface has not been directly established.

These findings provide a biological rationale for investigating PRP in patients with OS-related endometrial dysfunction, but redox and mitochondrial modulation should currently be regarded as hypothesis-generating mechanisms rather than established reproductive effects of PRP.

### 4.6. PRP EVs and microRNA Signaling

EVS, including exosomes and microvesicles, have emerged as potential mediators of the biological effects of PRP, extending its functional influence beyond the actions of soluble growth factors. These vesicles are generated following platelet activation and contain bioactive molecules, including proteins, lipids, messenger RNA, and microRNAs (miRNAs), that can be transferred to recipient cells [122,123,124].

PRP-derived EVs can influence gene expression in recipient cells through epigenetic and post-transcriptional mechanisms [86,87]. In reproductive studies, EVs and miRNAs have been implicated in processes such as trophoblast invasion, angiogenesis, implantation, and immune tolerance [86,87]. However, these lines of evidence should not be interpreted as demonstrating that PRP-derived EVs directly regulate these processes in the human endometrium or in RPL.

The miR-200 family regulates epithelial–mesenchymal transition, a process essential for trophoblast invasion, whereas members of the miR-21 and miR-30 families have been implicated in the regulation of cell proliferation and endometrial remodeling [100,125,126]. Additionally, the transfer of regulatory RNAs has been implicated in endometrial receptivity and implantation-related signaling [127,128]. Nevertheless, direct evidence that miRNAs delivered specifically by intrauterine PRP regulate HOXA10, LIF, or other implantation-related genes in women with RPL is currently lacking.

Experimental and extrapolated evidence further suggests that PRP-derived EVs may modulate immune responses by altering cytokine production [10,129] and may participate in the regulation of mitochondrial function and OS [130,131]. These effects, however, have not been prospectively demonstrated at the maternal–fetal interface following intrauterine PRP administration in women with RPL.

Compared with soluble factors, EVs may provide more stable and targeted signaling because their lipid bilayer protects their cargo from degradation and facilitates uptake by target cells [132]. Thus, PRP-derived EVs represent a plausible emerging mechanism through which platelet-derived signals could influence reproductive tissues. However, their specific contribution to implantation, pregnancy maintenance, and RPL remains predominantly experimental and hypothesis-generating [133,134].

Collectively, these advanced signaling mechanisms provide a rationale for further mechanistic investigation of PRP but should not yet be considered established therapeutic mechanisms in RPL. Further studies directly characterizing PRP-derived EVs, their miRNA cargo, and their effects in human endometrial tissues and well-defined RPL populations are required.

## 5. Applications of PRP in RPL and ART

The clinical application of PRP in RPL is an emerging area of reproductive medicine. Although there is a biologically plausible mechanistic rationale for its investigation, clinical evidence specifically in RPL populations remains limited and is derived primarily from observational studies, pilot trials, and heterogeneous cohorts [89,104].

Importantly, a substantial proportion of the evidence derives from studies conducted in related conditions, particularly RIF and thin endometrium. While these conditions share overlapping biological mechanisms, especially impaired endometrial receptivity, they represent distinct clinical entities with different primary endpoints. Therefore, clinical evidence is presented separately below to avoid inappropriate extrapolation [30,31,89,135].

### 5.1. PRP in RPL

Direct clinical evidence evaluating intrauterine PRP specifically in women with RPL remains very limited. Among the studies identified, Nazari et al. conducted a randomized controlled trial in women with unexplained RPL undergoing IVF [136]. In this study, 63 women were randomized, although only 40 completed the trial. Clinical pregnancy occurred in 35% of women receiving PRP compared with 20% of controls, while live birth occurred in 15% versus 0%, respectively; however, these differences were not statistically significant. The small sample size, substantial attrition, and single-center design limit the certainty and generalizability of these findings.

Beyond this trial, robust clinical evidence specifically evaluating PRP in well-defined RPL populations is lacking. Consequently, pregnancy rates reported in studies of RIF, thin endometrium, or mixed infertility populations should not be considered direct evidence of efficacy in RPL. Although these studies may provide supportive information regarding the effects of PRP on implantation and endometrial function, they address clinically distinct populations and are considered separately below.

Notably, current RPL-specific evidence does not establish that PRP reduces the risk of subsequent miscarriage. Miscarriage and ongoing pregnancy have not been adequately evaluated across dedicated RPL trials, and the available evidence is insufficient to determine whether PRP influences pregnancy maintenance after implantation [88,104,136]. Therefore, improvements in implantation or clinical pregnancy observed in other reproductive populations should not be interpreted as evidence of miscarriage prevention in women with RPL.

The most clinically relevant outcome, live birth rate, remains insufficiently studied in RPL populations. In the available RPL-specific randomized trial, live birth occurred in 15% of women receiving PRP and in none of the controls, but this difference was not statistically significant [136]. Thus, there is currently insufficient direct evidence to conclude that PRP improves live birth in women with RPL. Live birth findings from RIF, thin-endometrium, or mixed infertility cohorts represent indirect evidence and cannot establish efficacy for RPL.

Methodological limitations further weaken the strength of the available evidence. These include small study populations, lack of randomization, variability in outcome definitions, and concurrent use of additional treatments such as hormonal support, anticoagulants, or immunomodulatory therapies. Moreover, heterogeneity in PRP preparation protocols, including differences in platelet concentration, leukocyte content, activation methods, and administration timing, limits reproducibility and comparability across studies [57,89,99].

Overall, the direct clinical evidence is currently insufficient to support a beneficial effect of PRP on miscarriage or live birth in women with RPL. Evidence from RIF and thin-endometrium populations remains valuable for understanding the potential endometrial and implantation-related effects of PRP but should be regarded as indirect and hypothesis-generating in the context of RPL.

### 5.2. Indirect Evidence from RIF

A substantial portion of the clinical evidence supporting PRP use in reproductive medicine derives from studies in patients with RIF [30,89,135]. In these studies, PRP is typically administered intrauterinely prior to embryo transfer in assisted reproductive technology (ART) cycles [137,138,139,140].

Several studies have reported improvements in implantation rates and clinical pregnancy outcomes following PRP administration in women with RIF [137,138,139,140,141,142]. For example, Russell et al. reported improvements in endometrial thickness, clinical pregnancy, and live birth compared with patients’ previous cycles, although the retrospective design, absence of a concurrent control group, and mixed RIF/thin-endometrium population limit interpretation [137]. Enatsu et al. similarly reported higher clinical pregnancy rates following PRP in women with RIF [138], whereas Ban et al. observed higher clinical pregnancy and live birth rates in a retrospective controlled RIF cohort [139]. However, other studies have produced less consistent findings, including the randomized trial by Allahveisi et al., which found no significant improvement in biochemical or clinical pregnancy rates [141]. These reported effects have been attributed to enhanced endometrial receptivity, improved local vascularization, and modulation of the endometrial immune environment [57,77,135]. More recently, a 2026 meta-analysis of randomized controlled trials evaluating intrauterine PRP in women with RIF reported improvements in several reproductive outcomes. However, these findings remain indirect in the context of RPL and cannot establish an effect on miscarriage prevention or live birth in women with RPL [143].

While these findings are encouraging, their relevance to RPL remains indirect. RIF and RPL represent distinct clinical conditions, with implantation failure and pregnancy loss reflecting different underlying biological processes. Therefore, although RIF studies provide supportive clinical and mechanistic evidence regarding the potential effects of PRP on implantation, their findings cannot be directly extrapolated to RPL, particularly with regard to miscarriage reduction or live birth outcomes [89,104,135].

### 5.3. Indirect Evidence from Thin Endometrium

PRP has also been extensively studied in patients with thin endometrium, where effects on endometrial growth and regeneration have been more consistently reported. In these populations, intrauterine PRP administration has been associated with increases in endometrial thickness, often from <7 mm to ≥7–8 mm, enabling embryo transfer in cycles that might otherwise have been canceled [66,67,137,144,145].

These findings suggest that one of the most consistently reported clinical effects of PRP is an improvement in endometrial parameters, supporting its potential role as a regenerative therapy targeting endometrial function [31,57,89,101]. More recent evidence from a multicenter observational cohort study of 280 embryo-transfer cycles demonstrated a greater increase in endometrial thickness following PRP treatment; however, PRP was not independently associated with biochemical pregnancy, clinical pregnancy, ongoing pregnancy, or live birth [146].

Improvements in endometrial thickness have also been accompanied in some studies by higher clinical pregnancy rates in ART settings [66,67,137,144,145]. However, these reproductive outcomes have not been uniformly demonstrated across studies, and the populations studied were not selected on the basis of RPL. A recent systematic review and meta-analysis restricted to randomized controlled trials suggested a potential improvement in clinical pregnancy following PRP or plasma rich in growth factors in women with thin endometrium; however, the certainty of evidence was very low, underscoring the need for larger and methodologically robust trials [147].

Similarly, a recent retrospective matched cohort study of 620 embryo-transfer cycles reported higher live birth rates following intrauterine PRP compared with matched controls (26.5% vs. 17.1%), with the association persisting after multivariable adjustment [148]. However, given the observational design, baseline differences between groups, and the absence of an RPL-specific population, these findings remain indirect evidence for RPL.

Nevertheless, as with RIF, these results should be interpreted cautiously when applied to RPL populations. While thin endometrium may contribute to implantation failure, it does not account for the full spectrum of mechanisms underlying RPL, particularly those related to placentation and early pregnancy maintenance [2,4,15,68,104,107]. Accordingly, improvements in endometrial thickness, implantation, clinical pregnancy, or live birth in thin-endometrium or other embryo-transfer cohorts should be regarded as indirect evidence and cannot be assumed to translate into a reduction in miscarriage or an improvement in live birth rates in women with RPL.

### 5.4. Limitations of Cross-Population Extrapolation

A major limitation of the current evidence base is the frequent extrapolation from RIF and thin endometrium populations to RPL. Although these conditions may share underlying mechanisms, particularly at the level of endometrial receptivity, they differ fundamentally in clinical presentation and outcome measures [66,89,104].

RIF is primarily characterized by failure of embryo implantation, whereas RPL involves failure of pregnancy maintenance after implantation has occurred. Consequently, therapeutic effects observed in RIF or thin endometrium, such as improved endometrial thickness or implantation rates, cannot be assumed to translate into reductions in miscarriage or improvements in live birth rates [2,4,30,31,68,89,135].

This distinction is critical when interpreting the available literature. Overall, direct high-quality evidence supporting the efficacy of PRP in strictly defined RPL populations remains limited, and current conclusions regarding miscarriage and live birth should therefore be based primarily on RPL-specific evidence rather than extrapolated from adjacent reproductive conditions [89,104,136].

Overall, the available clinical evidence supporting PRP in RPL is of limited certainty. Direct RPL evidence is based on small studies with limited statistical power and insufficient live-birth data, while most supportive findings derive from heterogeneous RIF, thin endometrium, or mixed infertility populations and frequently concern intermediate outcomes such as endometrial thickness, implantation, or clinical pregnancy. Additional limitations include non-randomized study designs and substantial heterogeneity in PRP preparation and administration protocols. Accordingly, these intermediate outcomes should not be interpreted as evidence of reduced miscarriage or increased live birth in women with RPL.

At present, the available data do not support the routine or indiscriminate use of PRP in RPL. Instead, its application should be considered experimental rather than established, with any potential clinical benefit in biologically selected RPL subgroups remaining to be confirmed in well-designed randomized controlled trials [89,99,104].

To improve clarity and facilitate comparison across studies, the key clinical studies evaluating PRP in RPL and related reproductive conditions are summarized in Table 2. The studies are categorized according to clinical population to distinguish direct RPL evidence from indirect evidence derived from RIF and thin endometrium cohorts.

## 6. Clinical Implications and Positioning of PRP in RPL

Among the pathogenic mechanisms implicated in RPL, reduced endometrial receptivity, immune dysregulation, impaired angiogenesis, and OS represent biologically plausible biological targets for investigation with PRP [15,23,26]. The available mechanistic evidence suggests that PRP may exert regenerative and immunomodulatory effects with the potential to influence endometrial function and promote a more favorable maternal–fetal interface [26,57,99].

Early clinical studies have also reported encouraging improvements in endometrial thickness, implantation, and clinical pregnancy rates. Nevertheless, the current evidence remains limited by small sample sizes, methodological heterogeneity, and the predominance of observational study designs [89,135,136]. Consequently, PRP should currently be regarded as an experimental intervention rather than an established treatment for RPL [89,99,104].

Importantly, the biological mechanisms discussed throughout this review suggest that the proposed effects of PRP are primarily relevant to abnormalities of the endometrium and maternal–fetal interface. This observation supports a selective rather than indiscriminate approach to future clinical investigation, emphasizing the importance of biologically guided patient selection [31,57,89,101].

Although PRP is an autologous product and is therefore expected to carry a low risk of immunogenicity, allergic reactions, or transmission of infectious agents [149,150,151], this favorable procedural safety profile should not be interpreted as evidence of established reproductive safety. Major adverse events have not been commonly reported following intrauterine administration in the available reproductive studies [27,152]; however, these studies are generally small, adverse-event reporting is inconsistent, and follow-up is insufficient to detect uncommon or delayed complications. Importantly, data on maternal, fetal, neonatal, and long-term offspring safety remain limited or absent. Specific obstetric and neonatal outcomes, including preterm birth, hypertensive disorders of pregnancy, fetal growth abnormalities, congenital anomalies, and neonatal morbidity, have also been inconsistently or insufficiently reported, precluding reliable conclusions regarding downstream pregnancy and neonatal safety. Furthermore, the high concentrations of growth factors contained in PRP, including VEGF, TGF-β, and PDGF, raise theoretical concerns regarding excessive angiogenesis or abnormal trophoblast invasion during early placentation [153,154]. These concerns are biologically plausible but have not been demonstrated as adverse outcomes in human clinical studies of intrauterine PRP. Accordingly, adequately powered prospective studies with systematic maternal, obstetric, fetal, neonatal, and long-term offspring follow-up are required before the reproductive safety of intrauterine PRP can be established.

The clinical implementation of PRP is further complicated by substantial variability in preparation and administration protocols. Conventional PRP, leukocyte-poor PRP, plasma rich in growth factors (PRGF), and other platelet-derived preparations may differ in platelet and leukocyte concentrations, activation procedures, and resulting biological composition and should therefore not be considered interchangeable. Differences in centrifugation procedures, platelet concentration, leukocyte content, activation methods, injection volume, number of administrations, timing and interval before embryo transfer, and route of delivery may influence the biological composition, tissue exposure, and clinical effects of these preparations [155]. In reproductive medicine, both intrauterine infusion and injectable approaches have been described, further contributing to protocol heterogeneity [155]. Moreover, incomplete reporting of these parameters in the existing clinical literature limits comparison across studies and may contribute to variability in reported outcomes. Standardization and transparent reporting of platelet-derived preparations and administration protocols represent prerequisites for meaningful comparison across future studies.

Given the investigational nature of PRP, careful patient counseling and shared decision-making are essential. Women experiencing recurrent miscarriage often face considerable psychological distress and may therefore be particularly vulnerable to pursuing unproven interventions [3,27,155]. Clinicians should clearly communicate the current uncertainty regarding efficacy, the lack of established long-term reproductive safety, the experimental status of PRP, potential risks, and available evidence-based therapeutic alternatives. Ethical concerns also arise from the increasing commercialization of PRP within reproductive medicine, particularly when offered outside appropriately designed clinical research settings.

### Patient Selection and Clinical Positioning of PRP

Current evidence does not support routine administration of PRP to women with RPL. Given the limited evidence regarding efficacy, live birth outcomes, and reproductive safety, PRP should currently be considered an investigational intervention in this population and should preferably be evaluated within appropriately designed prospective clinical trials. From a hypothesis-generating perspective, its biological rationale may be greatest in carefully selected patients with evidence of endometrial or maternal–fetal interface dysfunction [26,89,104,136].

Potential populations for future investigation may include women with unexplained RPL accompanied by impaired endometrial receptivity, thin endometrium, defective angiogenesis, persistent inflammatory activation, immune dysregulation, or increased oxidative stress. Related populations, including women with RIF or abnormal expression of endometrial receptivity markers, may also provide indirect evidence for investigating the biological effects of PRP, but should not be considered equivalent to women with RPL [15,23,30,31,57,89,135]. Notably, RPL itself represents a heterogeneous clinical phenotype, and any potential effect of PRP may not be uniform across affected women. Biologically distinct populations may include women with unexplained RPL, pregnancy loss despite euploid embryo transfer, RPL following assisted reproduction, RPL accompanied by thin endometrium, and cases characterized by suspected abnormalities of endometrial receptivity or early placentation. PRP may theoretically be more relevant to subgroups in which endometrial or maternal–fetal interface dysfunction contributes to pregnancy loss; however, these subgroups have not been prospectively evaluated as modifiers of PRP response, and current evidence does not support subgroup-specific treatment selection.

The reproductive stage at which PRP might exert a clinically relevant effect also remains uncertain. Its proposed actions could theoretically influence implantation, early placentation, and/or subsequent pregnancy maintenance, but current evidence does not establish which of these processes, if any, is predominantly affected in women with RPL.

Conversely, patients with clearly established non-endometrial etiologies of RPL, including parental chromosomal abnormalities, uterine structural anomalies, endocrine disorders, thrombophilia, or other well-defined systemic conditions, are less biologically plausible targets for PRP-based investigation, as these mechanisms fall outside its principal proposed biological targets. Endocrine factors, including thyroid-related disorders, should therefore be appropriately evaluated and managed as part of the broader assessment of reproductive dysfunction before considering experimental endometrial interventions [156]. In these patients, management should remain focused on evidence-based treatment of the underlying disorder [2,4].

**Figure 2:**
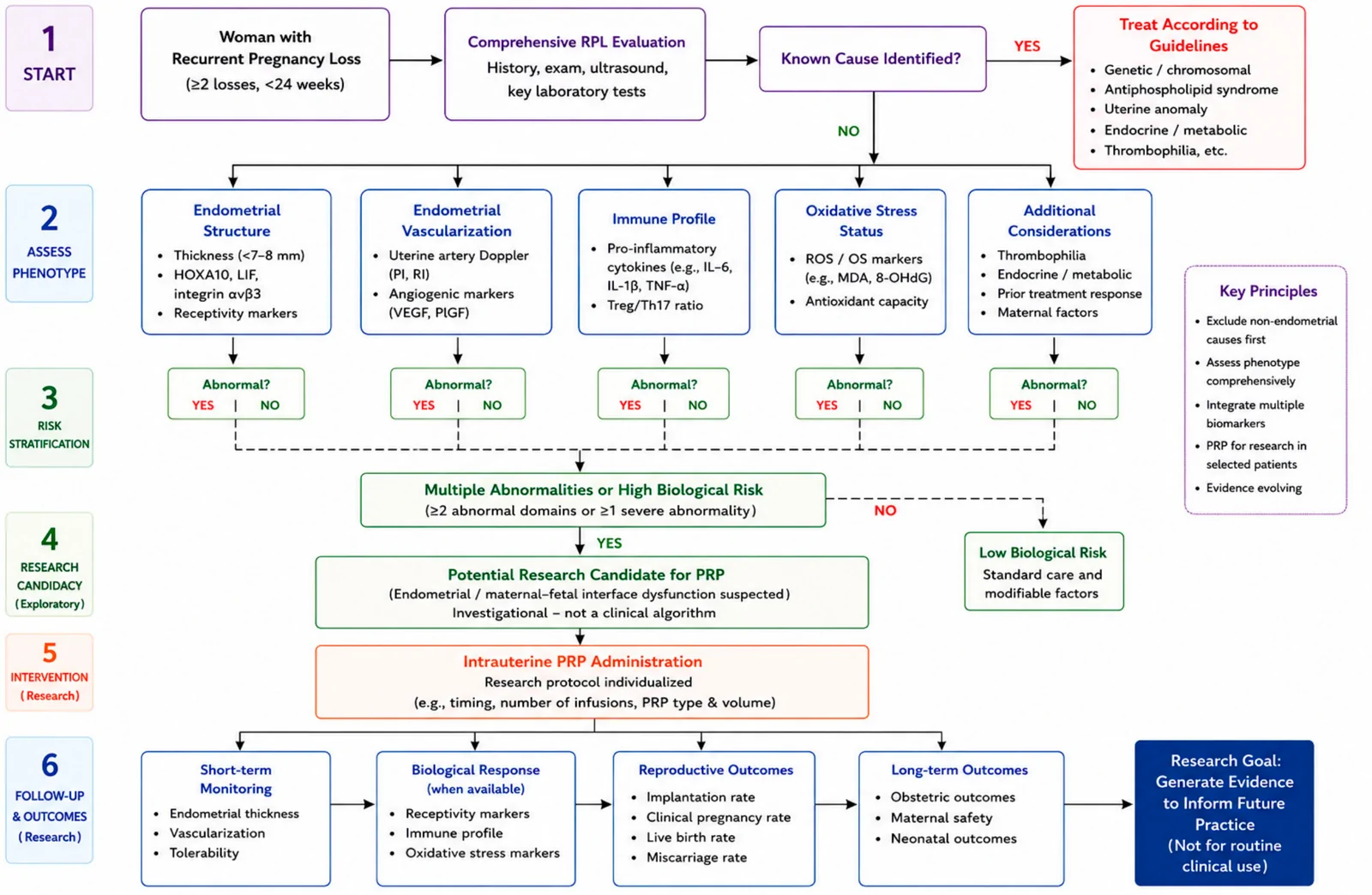
Hypothesis-generating research framework for evaluating the potential role of PRP in women with RPL. Biomarkers including HOXA10, LIF, integrin αvβ3, immune, angiogenic, and OS markers are investigational and have not been prospectively validated as predictors of PRP response or for PRP patient selection. This framework is intended to guide future biomarker-driven research and should not be interpreted as a clinical decision-making or treatment algorithm.

Building on the distinction between direct RPL evidence and indirect evidence from related reproductive populations, a hypothesis-generating research framework for biologically guided patient stratification is proposed in Figure 2. Following exclusion of established causes of RPL, women with suspected endometrial dysfunction could, in future research settings, be further evaluated using clinical characteristics together with emerging biological indicators of impaired receptivity, angiogenesis, immune regulation, and oxidative stress. These biomarkers remain investigational and have not been prospectively validated for PRP patient selection [38,50,104,125]. Accordingly, the framework is intended to guide future biomarker-driven research rather than current clinical decision-making.

## 7. Future Directions in Precision Reproductive Medicine

The future clinical application of PRP in recurrent pregnancy loss is unlikely to depend on broader empirical use, but rather on improved biological patient stratification [89,99,104]. Increasing recognition of RPL as a heterogeneous disorder supports the transition toward precision reproductive medicine, whereby treatment decisions are guided by individual molecular and clinical characteristics rather than by diagnosis alone [2,4,50,104].

### 7.1. Toward Biomarker-Guided Patient Stratification

One of the major limitations of current research is the absence of validated biomarkers capable of identifying women most likely to respond to PRP therapy. Although several molecular pathways have been implicated, these findings have not yet translated into clinically applicable tools for patient selection [89,99,104]. Markers associated with RPL or endometrial dysfunction include HOXA10, LIF, integrin αvβ3, VEGF, Treg/uNK profiles, inflammatory cytokines, and markers of OS [38,50,54,114,121]. However, an association with RPL or endometrial dysfunction should not be interpreted as evidence that these markers predict response to PRP. At present, they should therefore be regarded as candidate, hypothesis-generating biomarkers for prospective investigation rather than validated predictive biomarkers for PRP response. Advances in transcriptomics, proteomics, metabolomics, extracellular vesicle biology, and AI-assisted predictive models may further facilitate the investigation of biologically defined patient subgroups and potential treatment-response signatures. These approaches remain investigational and require prospective validation before they can inform PRP patient selection [38,104,123,133].

For near-term clinical research, readily identifiable clinical characteristics, such as unexplained RPL, RPL following euploid embryo transfer, and coexisting thin endometrium, may provide pragmatic variables for patient stratification in prospective trials. In contrast, molecular and cellular markers, including HOXA10, LIF, integrin αvβ3, VEGF, Treg/uNK profiles, inflammatory cytokines, and OS markers, remain experimental and require prospective validation before they can be used to predict PRP response or guide patient selection.

A stepwise approach may therefore be proposed: (i) exclusion of established non-endometrial causes, (ii) assessment of clinical indicators of endometrial dysfunction, and (iii) evaluation of PRP as an experimental intervention within appropriately designed clinical studies. Crucially, this paradigm remains hypothesis-driven and should be interpreted with caution until prospectively evaluated [2,104].

### 7.2. Research Priorities

Several important challenges must be addressed before PRP can be incorporated into routine clinical practice. Future randomized controlled trials should adopt standardized PRP preparation protocols, including platelet concentration, leukocyte content, activation methods, administration timing, and dosing schedules [57,89,99]. Future trials should incorporate biological stratification of participants according to endometrial phenotype to better identify responsive patient subgroups, rather than relying solely on heterogeneous RPL populations [38,104].

Standardization of outcome definitions, particularly for implantation, miscarriage, and live birth, is equally important to facilitate comparison across studies. Future trials should prioritize live birth rate as the primary clinical endpoint while incorporating candidate mechanistic biomarkers to investigate treatment response and elucidate the biological effects of PRP [2,38,50,89]. Large multicenter collaborations will be essential to achieve adequate statistical power, improve generalizability, and validate biomarker-guided therapeutic strategies [89,99].

Ultimately, integrating standardized PRP protocols with prospective biological stratification may help determine whether PRP can evolve from an experimental adjunctive therapy into a precision medicine approach for defined subgroups of women with RPL. Future clinical trials should incorporate biomarker-based patient stratification to evaluate individualized treatment strategies and establish the role of PRP within precision reproductive medicine [89,99,104].

## 8. Conclusions

In summary, PRP targets several biological pathways implicated in RPL, including impaired endometrial receptivity, angiogenesis, immune imbalance, and oxidative stress, providing a plausible mechanistic rationale for its investigation in this setting. Studies in related RIF and thin-endometrium populations suggest potential improvements in endometrial function and implantation outcomes; however, these surrogate or ART-specific findings should not be extrapolated to pregnancy maintenance in RPL.

The current evidence base is limited by small sample sizes, heterogeneous study populations, and variability in PRP preparation protocols. Importantly, robust evidence demonstrating a reduction in miscarriage or an improvement in live birth rates in women with RPL is still lacking. Consequently, definitive conclusions regarding the clinical efficacy of PRP cannot yet be drawn.

Although major adverse events have not been commonly reported in the available studies, this does not establish reproductive safety. Existing studies are generally small and lack sufficient follow-up to evaluate uncommon or delayed adverse outcomes, while data on obstetric, fetal, neonatal, and long-term offspring outcomes remain limited or absent. At present, PRP should not be considered a standard treatment for RPL but rather an experimental intervention that requires further evaluation within appropriately designed clinical research settings.

Future research should prioritize well-designed, adequately powered randomized controlled trials incorporating standardized PRP preparation protocols, biologically stratified patient populations, and live birth as the primary clinical endpoint. Such trials should also incorporate systematic assessment of maternal, fetal, and neonatal safety outcomes with adequate follow-up. Beyond demonstrating efficacy, the future of PRP in recurrent pregnancy loss will depend on the identification of predictive biomarkers capable of guiding patient stratification. The specific contribution of the present review is to refine the precision-medicine framework for RPL by distinguishing direct RPL evidence from indirect evidence derived from RIF, thin-endometrium, and other reproductive populations and by integrating this evidence with a hypothesis-generating framework for future biological stratification. Rather than expanding the empirical use of PRP, this framework is intended to guide future biomarker-driven research and the design of appropriately stratified clinical trials. Such an approach may ultimately determine whether PRP can evolve from an experimental regenerative intervention into a biologically targeted therapeutic strategy for prospectively defined RPL subgroups.

## Figures and Tables

**Table 2:** Summary of direct RPL evidence and indirect clinical evidence evaluating intrauterine PRP in related reproductive conditions. Abbreviations: RPL, recurrent pregnancy loss; RIF, recurrent implantation failure; EMT, endometrial thickness; PRP, platelet-rich plasma; PRGF, plasma rich in growth factors; FET, frozen embryo transfer; HRT, hormone replacement therapy; LBR, live birth rate; NR, not reported. PRP preparation and administration characteristics are presented as reported in the original studies; several studies did not provide complete information regarding platelet concentration, leukocyte content, activation method, or other preparation parameters.

Study	Design	Sample Size	Clinical Category	Population	PRP Preparation and Administration Protocol	Reproductive Outcomes	Major Limitations
Nazari et al. [136]	RCT	63 randomized; 40 completed	RPL—Direct evidence	Women with unexplained RPL undergoing IVF	Intrauterine infusion of 0.5 mL autologous PRP, administered 48 h before embryo transfer	Higher clinical pregnancy rate (35% vs. 20%); live birth observed only in PRP group (15% vs. 0%), not statistically significant	Small sample size; high dropout rate; lack of statistical significance; single-center study
Russell et al. [137]	Retrospective cohort study	85 patients (133 cycles)	RIF/thin endometrium—Indirect evidence	Women with RIF (56.5%), thin endometrium (27%), or both	Intrauterine PRP (0.5–0.75 mL) prepared from 21 cc blood using 2-step centrifugation; administered prior to FET (1 infusion per cycle in most cases)	Significant increase in endometrial thickness (~+1 mm); higher clinical pregnancy rate (37% vs. 20%); higher live birth rate (19% vs. 2%) compared to prior cycles	Retrospective design; no control group; within-patient comparison; mixed population; indirect relevance to RPL
Enatsu et al. [138]	Retrospective cohort study	54 patients/54 embryo transfer cycles	RIF/thin endometrium—Indirect evidence	Women with RIF (≥2 failed ET cycles), including thin endometrium (<8 mm) and unexplained RIF	Intrauterine PRP infusion prior to embryo transfer (FET cycles with high-quality blastocysts)	Higher clinical pregnancy rate (50% vs. 9.6% in prior cycles); higher hCG positivity (57.4% vs. 27.2%); no significant improvement in EMT	Retrospective design; no control group; within-patient comparison; small sample size; mixed population; indirect relevance to RPL
Ban et al. [139]	Retrospective cohort (with control group)	118 patients (64 PRP/54 control)	RIF—Indirect evidence	Women with RIF undergoing FET cycles	Intrauterine infusion of leukocyte-poor PRP prior to embryo transfer	Higher β-hCG positivity (57.8% vs. 38.9%); higher clinical pregnancy rate (45.3% vs. 24.5%); higher LBR (42.2% vs. 18.5%); no difference in miscarriage rate	Retrospective design; non-randomized; potential selection bias; no RPL population; indirect applicability to miscarriage outcomes
Castells et al. [144]	RCT with retrospective follow-up	22 patients (13 PRGF/9 control)	Thin endometrium—Indirect evidence	Women with very thin endometrium (≤5 mm) undergoing hormone-prepared FET cycles	Intrauterine instillation of PRGF, three infusions combined with estrogen therapy	Greater increase in EMT in PRGF group (+1.30 mm vs. +0.58 mm); some patients reached ≥7 mm; limited pregnancy and LBR	Very small sample size; limited number of embryo transfers; low statistical power; not RPL population; indirect relevance to miscarriage outcomes
Aghajanova et al. [145]	Single-arm prospective cohort study	46 patients (51 cycles)	Thin endometrium—Indirect evidence	Women with thin endometrium (EMT < 6 mm) and prior cancelled or failed FET cycles	Intrauterine PRP infusion prior to FET cycle	Significant increase in EMT (from 4.0 mm to 7.1 mm); 64.7% achieved ≥7 mm; clinical pregnancy rate 54.2%; live birth achieved in subset of patients	Single-arm design; no control group; small sample size; selection bias; not RPL population; outcomes influenced by ART context
Zamaniyan et al. [140]	Prospective controlled trial	98 patients	RIF—Indirect evidence	Women with ≥3 failed high-quality embryo transfers undergoing FET cycles	Intrauterine infusion of 0.5 mL autologous PRP (4–6× platelet concentration) administered 48 h before embryo transfer	Higher clinical pregnancy rate (48.3% vs. 23.3%); higher ongoing pregnancy rate (46.7% vs. 11.7%); higher implantation rate (58.3% vs. 25%) compared to control	Non-randomized design; baseline differences between groups; no direct RPL population; outcomes limited to implantation/pregnancy, not miscarriage
Allahveisi et al. [141]	RCT	50 patients (25 PRP/25 control)	RIF—Indirect evidence	Women with RIF undergoing FET cycles	Intrauterine infusion of 0.5 mL autologous PRP 48 h before embryo transfer (control: Ringer solution)	No significant difference in chemical pregnancy (28% vs. 36%) or clinical pregnancy rates (28% vs. 24%) between groups	Small sample size; limited statistical power; no live birth data; no RPL population
Eftekhar et al. [149]	RCT	83 patients (40 PRP/43 control)	Thin endometrium—Indirect evidence	Women with poor endometrial response (<7 mm) undergoing FET cycles	Intrauterine infusion of 0.5–1 mL autologous PRP on day 13 of HRT cycle; repeated after 48 h if needed	Significant increase in endometrial thickness (8.67 mm vs. control); higher implantation and clinical pregnancy rates	No live birth data; limited follow-up; not RPL population; ART-specific setting
Safdarian et al. [142]	RCT	120 patients (60 PRP/60 control)	RIF—Indirect evidence	Women with RIF undergoing frozen-thawed embryo transfer	Intrauterine infusion of 0.5 mL autologous PRP 48 h before embryo transfer	Higher implantation rate (28% vs. 11.9%); higher clinical pregnancy rate (51.6% vs. 26.6%); higher LBR (58.3% vs. 28.3%); no difference in miscarriage rate	Single-center study; no RPL population; increased preterm delivery; findings not generalizable to miscarriage outcomes
Nayar et al. [66]	Prospective cohort study (non-randomized)	100 patients (70 PRP/30 control)	Thin endometrium—Indirect evidence	Women < 40 years with thin endometrium (EMT < 7 mm) undergoing FET cycles	Intrauterine PRP instillation on days 7, 9, and 11 of HRT cycle	Increased EMT; higher clinical pregnancy rate (35.7% vs. 10%) compared to control	Non-randomized design; potential selection bias; no live birth data; not RPL population; ART-specific setting
Peng et al. [67]	Retrospective cohort study (with control group)	220 patients (104 PRP/116 control)	RIF + thin endometrium—Indirect evidence	Women with RIF and thin endometrium	Intrauterine PRP perfusion prior to embryo transfer	Increased EMT; improved uterine blood flow (↓ PI/RI); higher implantation, clinical pregnancy, ongoing pregnancy, and LBRs compared to control	Retrospective design; non-randomized; mixed population; potential confounding; not RPL population
Alonso-Frías et al. [146]	Retrospective multicenter observational cohort study	280 embryo-transfer cycles	Thin endometrium—Indirect evidence	Women with thin endometrium (3–6.9 mm) undergoing embryo transfer in HRT cycles	Intrauterine autologous PRP instillation during HRT cycles	Greater increase in EMT with PRP (0.95 ± 0.99 vs. 0.16 ± 0.77 mm); no independent association with biochemical pregnancy, clinical pregnancy, ongoing pregnancy, or live birth	Retrospective observational design; potential residual confounding; PRP protocol details not provided in abstract; not an RPL population
Xiao et al. [148]	Retrospective matched cohort study	620 ET cycles (310 PRP; 310 controls)	RIF/thin endometrium/mixed—Indirect evidence	Women undergoing embryo transfer; PRP group included higher proportions with clinician-recorded RIF and other reproductive/uterine histories	Intrauterine PRP infusion before embryo transfer	Improved endometrial parameters; live birth 26.5% vs. 17.1% (adjusted RR 1.55, 95% CI 1.12–2.15); higher clinical and ongoing pregnancy rates	Retrospective observational design; baseline clinical differences despite matching; potential residual confounding; heterogeneous indications for PRP; not an RPL-specific population

## Data Availability

No new data were created or analyzed in this study. Data sharing is not applicable to this article.
